# CDK5RAP2 interaction with components of the Hippo signaling pathway may play a role in primary microcephaly

**DOI:** 10.1007/s00438-016-1277-x

**Published:** 2016-12-21

**Authors:** Salil K. Sukumaran, Maria Stumpf, Sarah Salamon, Ilyas Ahmad, Kurchi Bhattacharya, Sarah Fischer, Rolf Müller, Janine Altmüller, Birgit Budde, Holger Thiele, Muhammad Tariq, Naveed Altaf Malik, Peter Nürnberg, Shahid Mahmood Baig, Muhammad Sajid Hussain, Angelika A. Noegel

**Affiliations:** 1 Institute of Biochemistry I, Medical Faculty, University of Cologne, Joseph-Stelzmann-Str. 52, 50931 Köln, Germany; 2 Center for Molecular Medicine Cologne (CMMC), University of Cologne, 50931 Köln, Germany; 3 Cologne Excellence Cluster on Cellular Stress Responses in Aging-Associated Diseases (CECAD), University of Cologne, 50931 Köln, Germany; 4 Cologne Center for Genomics (CCG), University of Cologne, 50931 Cologne, Germany; 5 Health Biotechnology Division, National Institute for Biotechnology and Genetic Engineering (NIBGE), Faisalabad, Pakistan

**Keywords:** Centrosome, MCPH, Hippo pathway, YAP/TAZ, MST1

## Abstract

Autosomal recessive primary microcephaly (MCPH) is characterized by a substantial reduction in brain size but with normal architecture. It is often linked to mutations in genes coding for centrosomal proteins; however, their role in brain size regulation is not completely understood. By combining homozygosity mapping and whole-exome sequencing in an MCPH family from Pakistan, we identified a novel mutation (XM_011518861.1; c.4114C > T) in *CDK5RAP2*, the gene associated with primary microcephaly-3 (MCPH3), leading to a premature stop codon (p.Arg1372*). CDK5RAP2 is a component of the pericentriolar material important for the microtubule-organizing function of the centrosome. Patient-derived primary fibroblasts had strongly decreased CDK5RAP2 amounts, showed centrosomal and nuclear abnormalities and exhibited changes in cell size and migration. We further identified an interaction of CDK5RAP2 with the Hippo pathway components MST1 kinase and the transcriptional regulator TAZ. This finding potentially provides a mechanism through which the Hippo pathway with its roles in the regulation of centrosome number is linked to the centrosome. In the patient fibroblasts, we observed higher levels of TAZ and YAP. However, common target genes of the Hippo pathway were downregulated as compared to the control with the exception of BIRC5 (Survivin), which was significantly upregulated. We propose that the centrosomal deficiencies and the altered cellular properties in the patient fibroblasts can also result from the observed changes in the Hippo pathway components which could thus be relevant for MCPH and play a role in brain size regulation and development.

**Electronic supplementary material:**

The online version of this article (doi:10.1007/s00438-016-1277-x) contains supplementary material, which is available to authorized users.

## Introduction

Autosomal recessive primary microcephaly (MCPH; MIM 251200) is a rare heterogeneous developmental congenital brain disorder characterized by a reduced occipitofrontal circumference of the head. Mutations in MCPH genes reduce the population of neurons in each of the six layers of the cerebral cortex during development leading to reduced thickness of the cerebral cortex. Patients with mutations at MCPH loci show moderate to severe microcephaly. Some of the notable phenotypes of microcephaly patients are a decrease in head size and brain volume, seizures and mental retardation, but no motor deficit (Kaindl et al. 2010). According to the OMIM data base, 16 genes have been identified as cause of MCPH. Several of the MCPH-associated proteins localize to centrosomes; however, there is still little information available relating to centrosomal mutations and brain size regulation. The centrosome is the main microtubule-organizing center in the cell and is involved in many different cellular processes, particularly during cell division, cell migration and differentiation (Bornens 2012; Conduit et al. 2015).

Cyclin-dependent kinase 5 regulatory subunit 2 (CDK5RAP2) is a pericentriolar structural component functioning in γ-tubulin ring complex (γTuRC) attachment and in the microtubule-organizing role of the centrosome (Kraemer et al. 2011; Fong et al. 2008, 2009). Mutations of *CDK5RAP2* are the cause of both primary microcephaly and Seckel syndrome (Bond et al. 2005; Yigit et al. 2015). CDK5RAP2 is a 215-kDa protein originally identified as CDK5 regulatory kinase 1 (CDK5R1)-binding protein. Near the N terminus it contains the γTuRC-binding site followed by an EB1-binding domain, the CDK5R1-interaction domain, a domain responsible for pericentrin binding and for Golgi complex association and several SMC domains along the molecule. It has many roles, among others it regulates mitotic spindle positioning, asymmetric centrosome inheritance, centriole replication, DNA damage signaling, and also has a spindle checkpoint function (Barr et al. 2010; Zhang et al. 2009; Barrera et al. 2010; Lizarraga et al. 2010).

A mouse mutant, *Hertwig’s anemia* (*an*) mouse, provided the first mechanistic link between the centrosome function of CDK5RAP2 and primary microcephaly. In this mouse, which suffers from severe hypoproliferative anemia and leukopenia and shows a high level of spontaneous aneuploidy in primary cultures of fetal cells, the underlying mutation was identified in *Cdk5rap2*. Similar to human the mice exhibited microcephaly with neurogenic defects including proliferative and survival defects in neuronal progenitors (Lizarraga et al. 2010). Phenotypes of the patients that result from *CDK5RAP2* mutations are sensorineural hearing loss, intellectual disability and a reduced occipital frontal head circumference (Pagnamenta et al. 2012; Issa et al. 2013). Therefore, from the human phenotype and the mouse mutant, an influence of CDK5RAP2 on the regulation of brain size during fetal development is apparent. Whether mutations in *CDK5RAP2* and the decrease in neuronal cell density are associated with altered signal transduction pathways is not really known although phosphorylation by LRRK1, a kinase that regulates the orientation of mitotic spindles, has been reported. This phosphorylation may affect the formation of the CDK5RAP2–γTURC complex (Hanafusa et al. 2015). We recently reported that CEP161, the CDK5RAP2 ortholog of *Dictyostelium discoideum*, binds to hippo-related kinase Hrk-svk and inhibits its kinase activity thereby presumably leading to inactivation of the pathway. We further found that it colocalizes with MST1 at the centrosome (Sukumaran et al. 2015).

The Hippo signaling pathway functions to restrict growth in adult tissues and modulates cell proliferation, differentiation, and migration in developing organs. Thus, it is a tumor-suppressive pathway. It primarily affects the number of cells produced whereas it has only minor effects on tissue patterning (Halder and Johnson 2011; Yu et al. 2015). The center of the Hippo pathway is formed by a kinase cassette consisting of two related serine/threonine kinases, mammalian STE20-like protein kinase 1 (MST1 and MST2), that are homologous to *D. melanogaster* Hippo (HPO), and large tumor suppressor 1 (LATS1) and LATS2 together with the adaptor proteins Salvador homologue 1 (SAV1) and MOB kinase activator 1A (MOB1A) and MOB1B (Udan et al. 2003; Harvey et al. 2003). They limit tissue growth by facilitating LATS1- and LATS2-dependent phosphorylation of the transcriptional activators Yes-associated protein (YAP) and transcriptional co-activator with PDZ-binding motif (TAZ) which promotes 14-3-3 binding resulting in their retention in the cytosol (Kanai et al. 2000).

YAP and TAZ function through regulation of the activity of several families of transcription factors such as Transcriptional Enhancer Factor Domain (TEAD) and Similar to Mothers Against Decapentaplegic (SMAD) family members. TEADs are key mediators of growth and presumably are responsible for the tumorigenic potential of YAP and TAZ. The genetic program that is regulated by these factors and promotes tissue growth is not well defined (Hong et al. 2005). Hippo signaling is also crucial for regulating the size of the mammalian liver; however, it does not appear to regulate the size or growth of other mammalian tissues to the same degree (Dong et al. 2007; Song et al. 2010).

In this study, we report the identification of a novel mutation in *CDK5RAP2* in an MCPH family. We further investigate a potential interaction of CDK5RAP2 with the Hippo signaling pathway and use patient-derived fibroblasts to study whether Hippo signaling is affected by the mutation. We identified MST1 as a CDK5RAP2 interaction partner and found that CDK5RAP2 has an impact on components of the Hippo signaling pathway such as YAP and TAZ. YAP/TAZ plays important roles in development in general and also in brain development as demonstrated in vertebrates (Piccolo et al. 2014). It was also shown that when YAP/TAZ is inhibited the expansion of neural progenitor cells is limited (Lavado et al. 2013). A role for MST1 at the centrosome and particularly in centriole formation has been shown previously (Hergovich et al. 2009). We find that MST1 knockdown has effects on centrosome nucleus distance, whereas the association of CDK5RAP2 with the centrosome appeared unperturbed. Based on our findings, we propose that aside from its role as a centrosomal component, CDK5RAP2 might have an additional role in the regulation of the brain size through its interaction with MST1 which impacts on the activity of the Hippo signaling pathway.

## Materials and methods

### Subjects

Approval of this study was obtained from the ethics review board of the Medical Faculty, University of Cologne and the National Institute for Biotechnology and Genetic Engineering in Faisalabad, Pakistan, according to the Declaration of Helsinki protocols. After getting consent from the parents, venous blood was obtained from both affected persons, parents and one from a normal individual of the MCP105 family.

### Linkage analysis

DNA was extracted from peripheral blood samples using standard methods. All the available individuals from the family were genotyped using highly polymorphic microsatellite markers spanning the regions of seven known MCPH loci. Later on, the identified homozygosity at the MCPH3 locus was corroborated by the genotyping of two affected individuals with the Axiom^®^ Genome-wide CEU Array according to the manufacturer’s protocols (Affymetrix, Santa Clara, CA). Assuming autosomal recessive mode of inheritance, full penetrance, consanguinity and a disease allele frequency of 0.0001, we performed linkage analysis as described previously (Hussain et al. 2013).

### Genomic DNA sequencing

One microgram DNA of affected individual VI-2 was used for whole-exome sequencing (WES). SeqCap EZ Human Exome Library v2.0 kit from NimbleGen (Roche NimbleGen Inc., Madison, WI 53719, USA) was used which needs a DNA probe for sequencing and the sample was run on an Illumina HiSeqTM 2000 sequencing instrument. The detailed procedure for WES carried out here was also described previously (Hussain et al. 2013). VARBANK database (https://varbank.ccg.uni-koeln.de) designed at the Cologne Center for Genomics (CCG) was used to filter data and to prioritize variants. Sanger sequencing was employed to sequence the targeted region of exon 27 of CDK5RAP2 harboring the mutation. Primer sequences to amplify exon 27 are enlisted in Supplementary Table 1.

### Cell culture and transfection experiments

HeLa and HEK293T cells were grown in Dulbecco’s modified Eagle’s medium (DMEM) supplemented with 10% fetal bovine serum, penicillin and streptomycin (100 μg/ml), l-glutamine and non-essential amino acids at 37 °C and 5% CO_2_. Transfection was done using Lipofectamine 2000 (Invitrogen) according to the manufacturer’s instructions (HeLa) or Polyethyleneimine (PEI, Polysciences, cat No 23966-2) for HEK293T cells. HeLa cells were used for immunofluorescence analysis, HEK293T cells for protein analysis. Transfected cells were incubated for 24–48 h post-transfection. A CDK5RAP2 plasmid was obtained from addgene (pRcCMV Cep215 (Nigg CW493), 40). It was used for generation of truncated versions and point mutations which were introduced by site-directed mutagenesis. The following mutant versions encoding shortened polypeptides were generated: CDK5RAP2-C corresponding to nonsense mutation c.246T > A, p.Y82* (residues 1–82) (Park et al. 2011), CDK5RAP2-C1 (residues 1–580) encompasses the γTuRC and SMC domains, CDK5RAP2-C2 (residues 1–1271) and CDK5RAP2-C3 (residues 1–1372) encompass the γTuRC, SMC and EB1 domain. GFP-MST1 and Flag-TAZ are described in Habbig et al. (2011); Flag-hnRNPF was obtained from Dr. Ping Li. MST1 knockdown was achieved in HeLa cells by siRNA using MST1 siRNA (Set I S25-911-05, SignalChem, Richmond, BC, Canada). RNAi transfection was performed using Interferin (Polyplus) as transfection reagent and siRNAs at a concentration of 1 to 10 nM. The protocol provided by the manufacturer was followed. 72 h after transfection cells were fixed or harvested. The efficiency of the knockdown was assessed by western blot analysis. For control, cells were treated with siRNA control oligos (Li and Noegel 2015).

### Patient cell culture and analysis

Primary fibroblasts were established from a biopsy of patient VI-2. The detailed procedure has been previously described (Hussain et al. 2013). The fibroblasts from patient (passages 3 and 4) and respective controls (passages 4 and 6) were cultured in Dulbecco´s Modified Eagle’s Medium (DMEM, PAA supplemented with 10% fetal bovine serum (FBS, Biochrom), l-Glutamine (PAN Biotech) and antibiotics (Penicillin/Streptomycin, PAN Biotech). Care was taken to carry out all experiments with cells at similar densities and passage number.

*Cell cycle and growth analysis* Cells were trypsinized and 5 × 10^5^ cells dissolved in DMEM were stored on ice. After incubation with Nuclear-ID™ Red DNA stain (Enzo Life Sciences), diluted 1:250, for 5 min at 37 °C, the cells were sorted immediately using the BD FACSAira III cell sorter (BD Bioscience USA). Cell sorting was carried out at the CMMC facilities. For analysis of growth, cells were trypsinized and counted.

*Cell size determination* For cell size determination, the cells were trypsinized and photos immediately taken with a microscope using a 10× objective. Diskus software was used for size measurements.

*Cell migration assay* For cell migration experiments, cells were seeded at equal densities in Ibidi culture inserts placed in eight-well Ibidi μ-slides. Next day, the inserts were removed and cells were allowed to migrate into the 500-μm gap generated by the insert and images were captured at 15-min intervals for 24 h with a Leica microscope (Leica DMI6000 B, LAS AF software version 2.0.2 build 2038) equipped with a camera (Hamamatsu) and using a 10×/0.25 NA dry objective and magnification of 1.6×. The slides with fibroblasts were placed in a humidified 5% CO_2_ atmosphere. For 37 °C warm air incubation the microscope and objectives were encased (heater and ventilation ON). Later, processing and analysis was performed using Image J ‘Manual Tracking’ and ‘Chemotaxis tool’. To study polarization, cells were fixed at the indicated time points and stained for Golgi and centrosome with 58 K Golgi protein (Abcam) and pericentrin-specific antibodies (Abcam), respectively. Golgi and centrosome polarity was determined for the first row of cells by drawing a wound-facing 120° sector on the cells. In general, the experiments were carried out at least three times or as indicated.

### RNA isolation and cDNA generation for quantitative RT-PCR analysis

Total RNA was extracted from cells grown in a monolayer in cell culture dishes with a kit following the instructions of the supplier (Promega, Heidelberg, Germany). First-strand cDNA synthesis was performed using M-MLV reverse transcriptase RNase H Minus-kit from Promega. Each sample for real-time RT-PCR analysis contained 200 ng of cDNA, SYBR Green Master Mix and 0.4 μM of each primer. The PCR amplification and real-time fluorescence detection were performed with the Opticon III instrument (MJ Research) using the QuantitectTM SYBR1 green PCR kit (Qiagen, Hilden, Germany). As quantification standard defined concentrations of annexinA7 cDNA were used for amplification. PCR amplification was carried out according to the manufacturer’s instruction and all PCR products were amplified in a linear cycle. GAPDH mRNA was employed as an internal standard, and expression of each gene was determined by RT-PCR and normalized against WT GAPDH mRNA levels. Data are the mean ± SD from three samples per group of four independent experiments. All primers are listed in Supplementary Table S1.

### Immunofluorescence analysis

For immunofluorescence, cells were grown on 12-mm coverslips and fixed with 3% paraformaldehyde (5 min, RT), followed by permeabilization with 0.5% Triton X-100 for 3 min (RT). Further steps were done as described (Hussain et al. 2013). Imaging was done by confocal laser scanning microscopy (Leica TCS-SP5). Images were processed using TCS-SP5 software. Antibodies against the following proteins were used: CDK5RAP2 (pAb, 06-1398, Millipore), anti-Myc (mAb 9E10), β-actin (mAb A2228, Sigma), α-tubulin (mAb YL1/2), γ-tubulin (mAb, T6557, Sigma), YAP/TAZ (mAb, 8418, Cell Signaling), MST1 (pAb, 3682, Cell Signaling), phospho-MST1/MST2 (pAb, 3681, Cell Signaling), GM130 (mAb, 618822, abcam), 58 K (mAb, ab27043, Abcam), pericentrin (pAb, ab4448, Abcam), γ-tubulin (mAb GTU-88, Sigma), Nesprin-1 (SpecII; (Taranum et al. 2012), BIRC5 (mAb, ab76424, Abcam), Ki67 as cell proliferation marker (mAb, M 7249, DAKO), anti-PH3 rabbit polyclonal IgG to stain mitotic cells (06-570, Upstate Biotechnology) and Cleaved Caspase-3 (ASP175)-specific polyclonal antibodies as apoptosis marker (No 9661, Cell signaling).

### Immunoprecipitation

For immunoprecipitation experiments, HEK293T cells were lysed in 10 mM Tris/HCl, pH 7.4, 50 mM NaCl, 0.5% NP40, protease inhibitor cocktail, 0.5 mM PMSF, 0.5 mM EDTA, 1 mM Benzamidine and incubated for 30 min at 4 °C (to ensure complete cell lysis) followed by a centrifugation step at 16,000 rpm for 10 min at 4 °C. The supernatants were either incubated with GFP-trap beads (ChromoTek, Martinsried, Germany) or with Flag-trap beads (Sigma, St. Louis, USA, Catalog Number A2220) for 2 h at 4 °C. GFP-trap beads were washed with a different wash buffer (10 mM Tris/HCl, pH 7.4, 50 mM NaCl, protease inhibitor cocktail (Roche), 0.5 mM PMSF, 0.5 mM EDTA, 1 mM Benzamidine). The beads were resuspended in SDS sample buffer, incubated at 95 °C for 5 min and the proteins were separated by SDS-PAGE and analyzed by western blotting using anti-GFP mAb K3-184-2 (Noegel et al. 2004) and Flag-tag specific rabbit polyclonal Ab (Sigma).

### Protein extraction from mammalian cells

Mammalian cells were trypsinized and washed with ice-cold 1x PBS plus protease inhibitor (DTT, Benzamidine and PMSF at 1 mM each). After centrifugation at 15,000 rpm at 4 °C the pellet was resuspended in lysis buffer (50 mM Tris/HCl, pH 7.5, 50 mM NaCl, 1% Nonidet P-40, protease inhibitor cocktail (PIC; Sigma) and further protease inhibitors DTT, Benzamidine and PMSF. The sample was denatured in SDS sample buffer at 95 °C for 5 min. The samples were used for SDS-PAGE and western blot analyses.

### Western blotting

For immunoblotting, equal amounts of total cell protein were separated by SDS-PAGE (12% and 3–12% acrylamide gradient gels) and subsequently transferred to nitrocellulose membranes. For protein transfer, semi-dry (45 min, 12 Volt) or wet blotting (overnight, 15 Volt) transfer was used. Subsequently, the membrane was blocked with 12.5 ml 1% blocking solution [1% milk powder in NCP buffer (NCP contains per liter, 10×: 12.1 g Tris, 87.0 g NaCl, 5.0 ml Tween 20, pH 8.0)] under constant shaking for 1 h. After blocking, the membrane was incubated with primary antibody solution for either overnight (+4 °C) or 1 h (RT). The membrane was washed three times with NCP for 15 min and incubated with the corresponding appropriate horseradish peroxidase-coupled secondary antibodies (1:10,000) for 1 h, and the membrane was washed three times with NCP. Antigen–antibody complexes were detected using the ECL western blotting detection solution. The protein bands were visualized using X-ray films. After imaging, the membrane was stripped with 0.2 M NaOH for 15 min. The stripped membrane was washed twice with NCP for 15 min. After washing the membrane, the membrane was blocked with blocking solution for 1 h at room temperature and used for antibody incubation.

### Data analysis and statistical evaluation

Unless otherwise stated, images were processed using ImageJ 1.47d (NIH), Adobe Photoshop CS version 8.0, and figures assembled using CorelDraw Graphics Suite X4. Data analyses and statistical evaluations were carried out using Microsoft Excel or GraphPad Prism; the number of independent experiments, mean values, standard errors, and *p* values (Student’s *t* test) are indicated in the figure legends.

## Results

### Clinical data

**Fig. 1:**
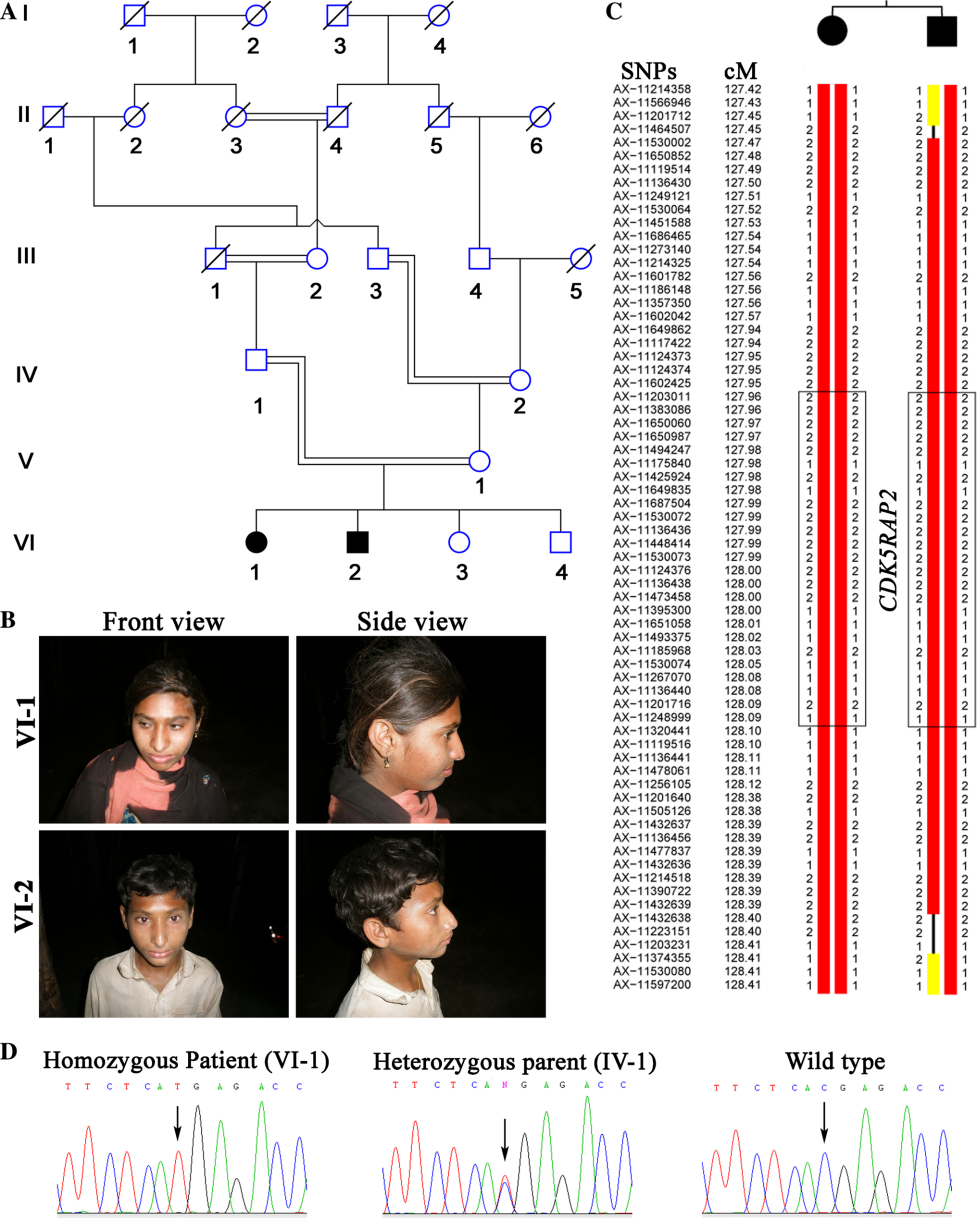
Characterization of patients and detection of the disease-causing mutation. **a** Pedigree of the MCP105 family. **b** Clinical features of individuals with a novel homozygous mutation of *CDK5RAP2* showing the typical signs of primary microcephaly. **c** Constructed haplotypes to depict the homozygous segment on chromosome 9 shared among the affected individuals. The homozygous region is surrounded by SNP markers AX-11201712 and AX-11374355 situated at the upstream and downstream regions of CDK5RAP2, respectively. The haplotype corresponding to each SNP marker is shown alongside the chromosomal region and the haplotype within the* boxed* region demonstrates the genomic region of CDK5RAP2. **d** Sanger traces of the relevant region of *CDK5RAP2* obtained with DNA samples of individual VI-1 and its heterozygous parent IV-1. The mutation c.4114C > T is absent from the wild-type trace

We ascertained a six-generation consanguineous Pakistani family with two affected individuals born to healthy consanguineous parents (Fig. 1a, b). At the time of clinical investigation, the affected female (VI-1) was 16 years old and had a head circumference of -5 SD compared to the average population of respective gender and age whereas her brother (VI-2) showed -9 SD at the age of 12 years. The patients had mild intellectual disability but had no other neurological symptoms or skeletal anomalies. Typical signs of Seckel syndrome like beaked nose and proportionate short stature were not seen in our patients (Yigit et al. 2015).

### Linkage analysis

All the available individuals from the family were genotyped using microsatellite markers flanking seven of the known MCPH loci. Homozygous alleles around the MCPH3 locus suggested linkage to this genomic region (data not shown). To verify these data, both affected members were genotyped using the Axiom^**®**^ CEU SNP Array (Affymetrix). Analysis of the genotyping data confirmed linkage on chromosome 9 (LOD score 2.4). Haplotype analysis revealed that a homozygous region on chromosome 9 spanning a 1-cM interval limited by the markers AX-11201712 and AX-11374355 (122,801,186-124,152,939; hg19) includes the MCPH3 locus (Fig. 1c, *CDK5RAP2* position is boxed).

### Identification of the disease-causing mutation in *CDK5RAP2*

To find the causal variant and exclude additional variants in other known MCPH-associated genes, the DNA sample of one affected individual (VI-2) was subjected to whole-exome sequencing (WES), which revealed a novel homozygous nonsense mutation in exon 27 of *CDK5RAP2* (c.4114C > T; p.Arg1372*; NM_018249.5). The mutation results in a truncated protein lacking the C-terminal CDK5R1 domain and the region responsible for pericentrin binding and Golgi association. The DNA variant was validated by Sanger sequencing. As expected, both patients were homozygous and the parents were heterozygous for the mutation (Fig. 1d). WES did not reveal homozygous or compound heterozygous variants in any one of the other known MCPH-associated genes.

### Characterization of CDK5RAP2 patient fibroblast cells

**Fig. 2:**
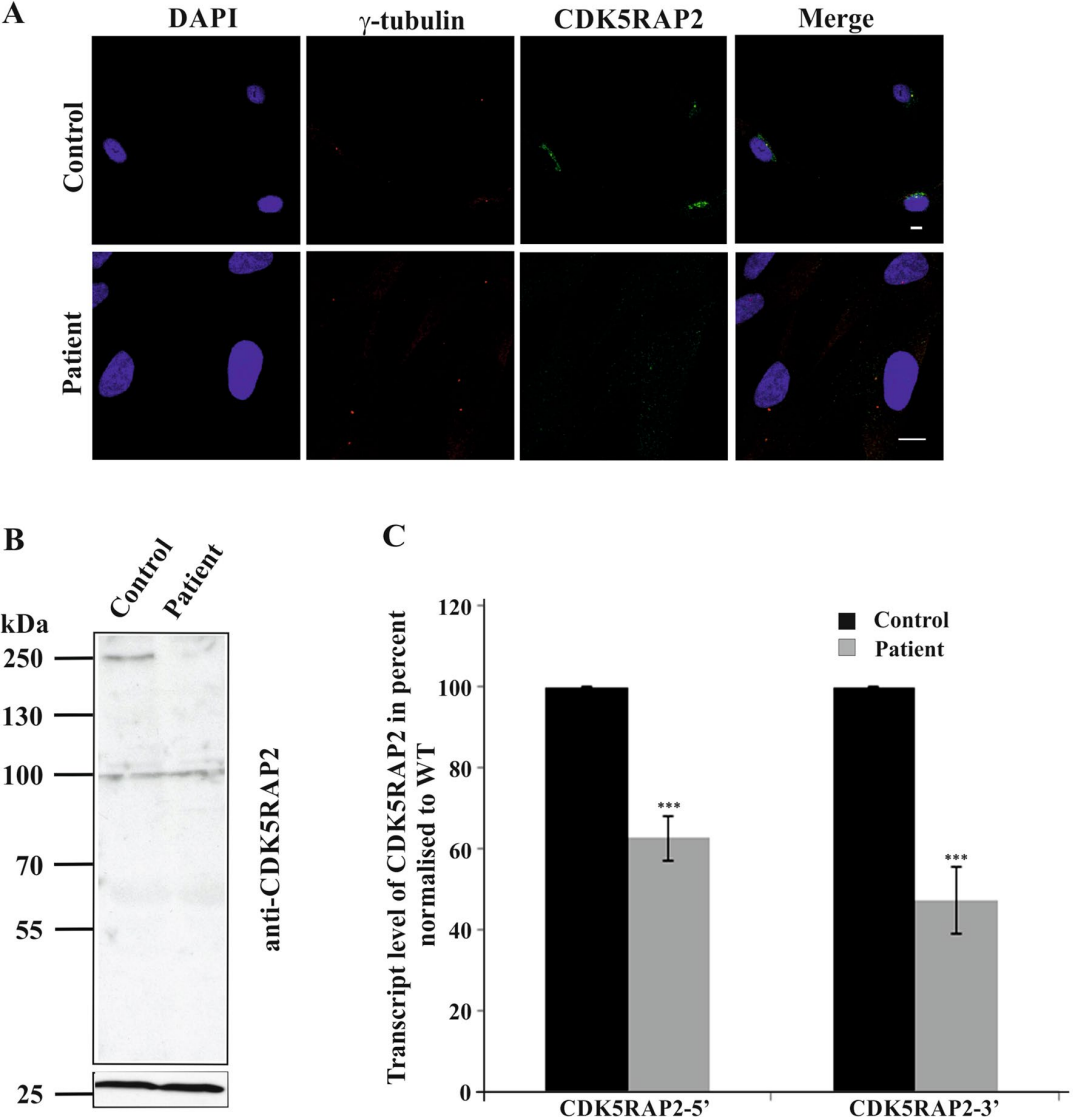
CDK5RAP2 is not detected in patient fibroblasts. **a** Localization of CDK5RAP2. CDK5RAP2 was detected with polyclonal antibodies, γ-tubulin detected with monoclonal antibodies was used as centrosomal marker and the nuclei were stained with DAPI. *Scale bar* 10 µm. **b** Detection of CDK5RAP2 in whole cell lysates. Whole cell lysates from control and patient fibroblasts were probed with polyclonal anti-CDK5RAP2 antibodies. The panel below represents the corresponding GAPDH levels. The signal at ~100 kDa is non-specific. **c** CDK5RAP2 transcript abundance in control and patient cells as analyzed by quantitative RT-PCR. Primers were derived from 5′ and 3′ regions of the cDNA sequence. The differences were significant (*P* < 0.001)

Immunofluorescence analysis with polyclonal antibodies specific for CDK5RAP2 showed in control fibroblasts a puncta staining in the vicinity of the nucleus which colocalized with γ-tubulin indicating its presence at the centrosome. Furthermore, CDK5RAP2 staining was also seen around the centrosome on tubule-like structures, which have previously been identified as Golgi membranes (Wang et al. 2010). In patient fibroblasts, we detected in some but not all cells a faint signal for CDK5RAP2 which colocalized with γ-tubulin. Furthermore, in cells exhibiting CDK5RAP2 staining, tubule-like structures were not observed (Fig. 2a). In western blot analysis, the CDK5RAP2-specific polyclonal antibodies detected in lysates from control fibroblasts a protein at ~250 kDa. The signal was rather faint and in the patient lysates the CDK5RAP2 amounts were below detection level emphasizing a reduction or absence of the protein. We also did not detect a truncated protein (expected at ~155 kDa) in the patient’s lysate (Fig. 2b). GAPDH was the loading control. In quantitative real-time PCR experiments (qRT-PCR) with primers derived from the 5′ and 3′ ends of the transcript, we found a significant reduction in the *CDK5RAP2* mRNA levels in the patient sample (5′ primer, ~60% of control; 3′ primer, ~42% of control) (Fig. 2c).

**Fig. 3:**
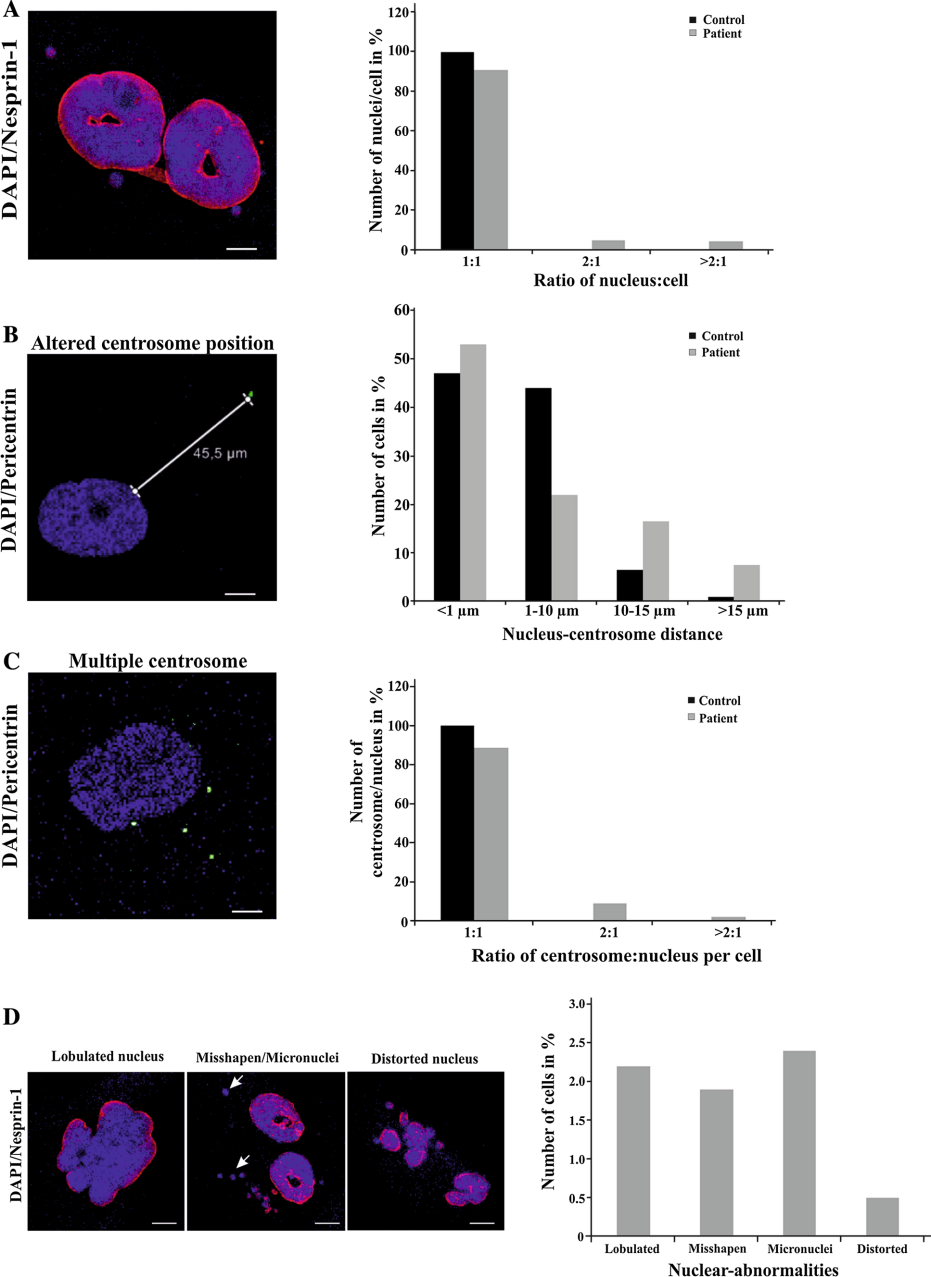
Nuclear aberrations and centrosome defects in patient cells. **a** Number of nuclei per cell. 450 control and 550 patient cells were evaluated. **b** Percentage of cells with the indicated nucleus–centrosome distance. The mean distance was 2.46 µm for control and 4.62 µm for patient cells. The difference was, however, not statistically significant. More than 100 cells were evaluated per strain. **c** Number of centrosomes per nucleus. ~450 cells were evaluated each. **d** Nuclear abnormalities as specified. More than 700 cells were evaluated. Percentage of cells with the abnormalities is shown in the *bar graphs* (**a**–**d**). Polyclonal antibodies (SpecII) against Nesprin-1 and pericentrin-specific antibodies were used to detect the nuclear envelope and the centrosome, respectively. DAPI was used to stain the nuclei. Arrows point to micronuclei. For the experiments passage 4 of the fibroblasts was used. *Scale bar* 5 µm

In further characterization of the patient-derived fibroblasts, we focused on nuclear and centrosomal aspects to understand the role of CDK5RAP2 in cell division and cell cycle. Control fibroblasts were mostly mononucleated, with their centrosome located close to the nucleus in a distance of <10 µm. By contrast, ~8% of the patient cells had multiple nuclei, which might be the result of a cytokinesis defect, and the centrosome nucleus distance was increased to >10 µm in 20% of the cells as compared to 4% in the control and >15 µm in 9% of patient cells (1% for control cells) (Fig. 3a, b). The centrosome–nucleus ratio was altered in 11% of the patient cells and 3% of the cells showed more than 2 centrosomes per nucleus (Fig. 3c). We also observed that the nuclei in ~7% of the patient cells had abnormal shapes. The abnormalities were classified as lobulated (2.3%), misshapen (1.8%), micronuclei (2.5%) and distorted (0.5%) (Fig. 3d). Nearly no such abnormalities were noted in control fibroblasts.

**Fig. 4:**
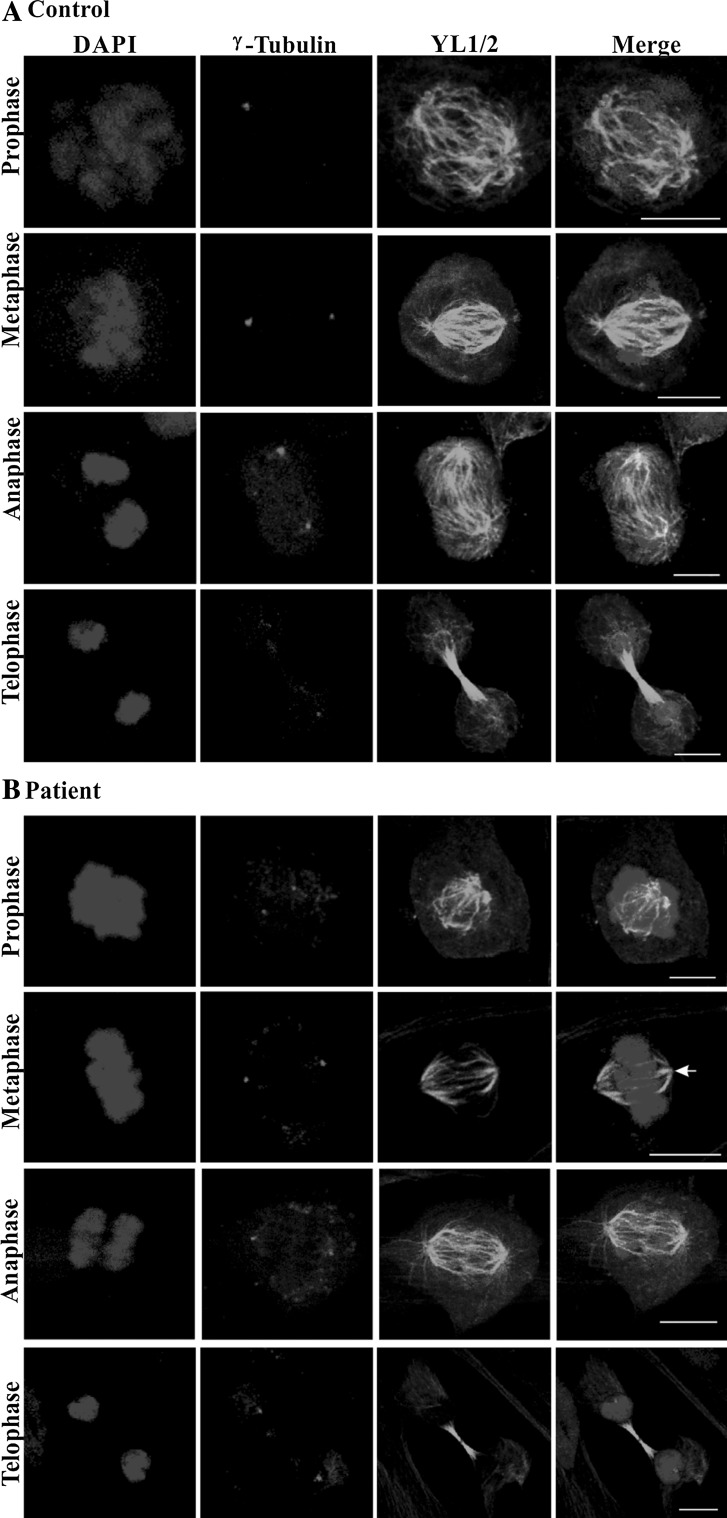
Mitotic stages in control (**a**) and patient cells (**b**). Confocal images of control and patient cells of the indicated mitotic stages are shown. Nuclei were stained with DAPI, centrosomes were detected with mouse mAb against γ-tubulin and microtubules were stained with rat mAb YL1/2. The *arrow* in B, metaphase, points to a spindle pole without prominent astral microtubules. *Scale bar* 10 µm

Analysis of the mitotic stages was performed in control and patient fibroblasts that were immunolabeled with mAb YL1/2 for staining of the tubulin network. The centrosome was detected with γ-tubulin-specific antibodies. Mutant and control fibroblasts progressed through mitosis in a similar fashion (Fig. 4a, b). The spindle fibers were less prominent in the patient cells and astral microtubules were not always visible (Fig. 4b, arrow, metaphase). However, this could be due to sample preparation as variable staining was also seen in control cells (see metaphase cell). Loss of astral microtubules had been observed upon knockdown of CDK5RAP2 but there are also conflicting results (Lucas and Raff 2007; Fong et al. 2008). By contrast, in interphase cells the tubulin staining appeared enhanced. Western blot analysis confirmed increased levels for γ-tubulin (relative intensities normalized to GAPDH levels: control, 0.546; patient, 1.173; *P*, 0.035; three independent experiments). At the transcript level, we also observed increased amounts in the patient (see below, Fig. 7e). We further performed FACS analysis to study cell cycle progression. Albeit we noted a tendency towards reduced length of the S and M phases in the mutant, the differences were not statistically significant (Table 1). Growth behavior was very similar and growth curves paralleled each other. Furthermore, in immunofluorescence analysis using markers for cell proliferation and apoptosis we did not observe differences (data not shown).

**Table 1:** Cell cycle progression of control and patient fibroblasts as studied by FACS analysis

	Control	Patient	*P* values
G0/G1 phase	78.16 ± 4.95	84.8 ± 3.59	0.3185
S phase	11.85 ± 3.01	8.80 ± 2.24	0.4473
M phase	6.98 ± 1.57	3.75 ± 0.34	0.0958

The data are mean values in percent derived from four independent experiments. The differences were not statistically significant (two-tailed *P* values)

**Fig. 5:**
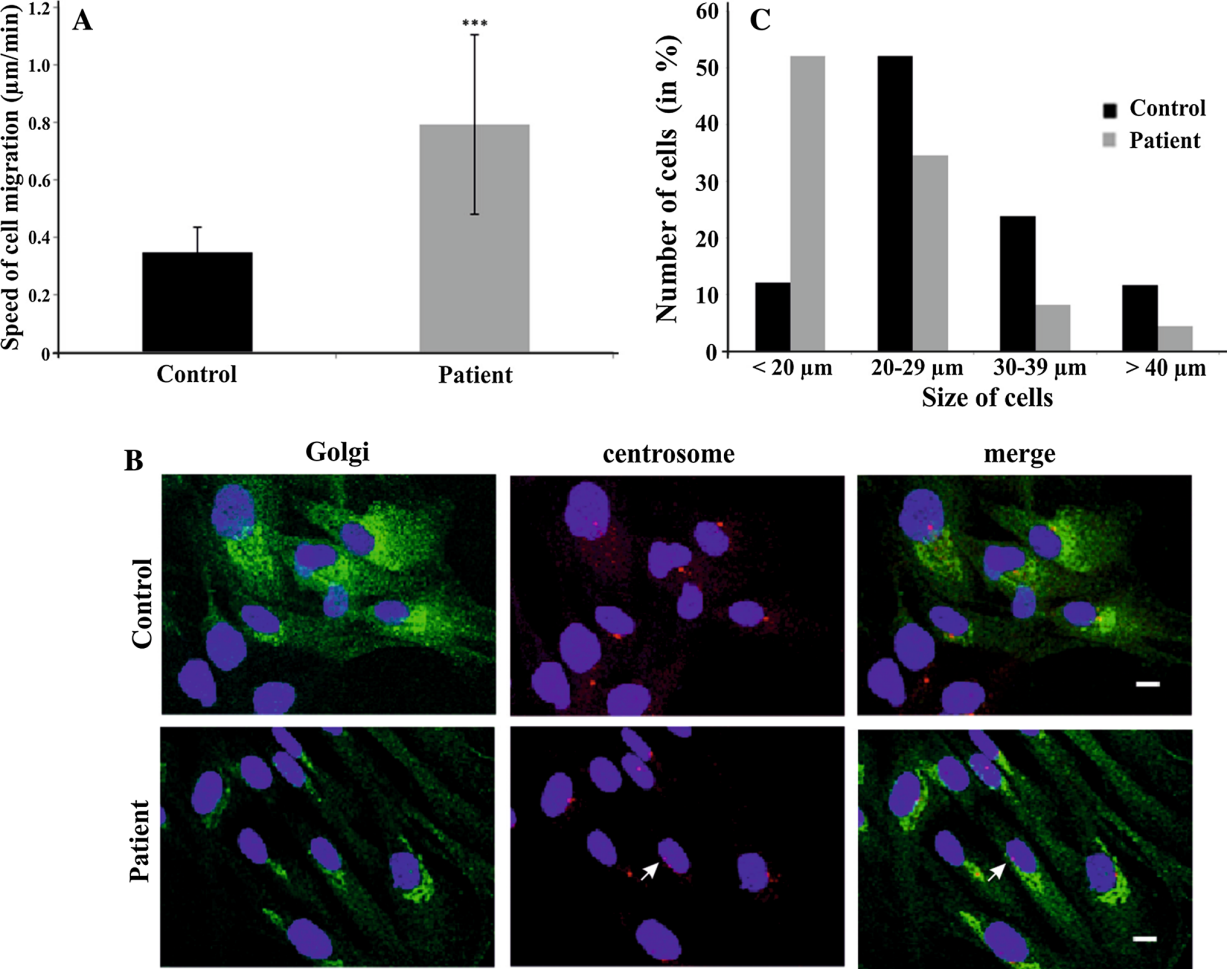
Cell migration, cell polarity and cell size analysis. **a** Analysis of speed during migration in µm/min (***P < 0.001). 32 cells each were analyzed. **b** Cell polarity analysis. Cells migrating into a scratch wound were fixed after 7 h of migration and stained for the Golgi (anti 58 K, mAb), centrosome (anti-pericentrin, pAb) and the nuclei (DAPI). The cells were migrating towards the* lower right* (location of the wound). The *arrow* points to a centrosome which is not colocalizing with the Golgi apparatus. *Scale bar* 10 µm. **c** Cell size of control and patient cells in micrometers. ~220 and 390 cells were analyzed, respectively

Since the centrosome regulates cell motility and cell polarity, we performed migration assays. The patient cells migrated with an average speed of 0.8 µm/min whereas the control cells traveled at an average speed of 0.35 µm/min (Fig. 5a). We also assayed the ability of the fibroblasts to reorient and migrate into a wound. In cell scratch assays, we found that the mutant cells closed the gap faster than the control which is presumably due to their increased migration speed. The gap closure also indicated that the cells were able to polarize and reorient. This was further tested by submitting the cells to immunofluorescence analysis seven hours after introduction of the scratch and staining for the centrosome and the Golgi apparatus which are both structures that reorient when cells polarize. We found that in the majority of the cells from control and patient the Golgi apparatus and the centrosome were located in front of the nucleus facing the gap. A comparable number of cells had their Golgi and centrosome in the back (Fig. 5b), and in some cells the centrosome and the Golgi were not colocalizing (12% in control, 29% in patient cells; Fig. 5b, arrow in patient sample).

A visual inspection of the patient fibroblasts had already indicated a smaller cell size. In further analyses, we confirmed this notion and found that patient cells were significantly smaller. Approximately, 50% of the cells had a diameter below 20 μm whereas in case of the control fibroblasts ~50% had a diameter between 20 and 29 µm (Fig. 5c). For these experiments, cells were trypsinized to obtain rounded cells as described in the Materials and Methods section. The smaller cell size was confirmed by determining the area where we measured for the majority of wild-type cells (42%) an area between 400 and 600 µm, whereas for the patient fibroblasts the majority of cells (52%) had an area of 200–300 µm (284 and 204 cells analyzed, respectively).

### CDK5RAP2 and the Hippo signaling pathway

In earlier work on CEP161, the *D. discoideum* ortholog of CDK5RAP2, we identified a link to Hippo signaling. CEP161 bound to the Hippo kinase Hrk-svk, and the N-domain of CEP161 inhibited the kinase activity. Overexpression of CEP161 resulted in reduced growth, defects in development and further deficiencies (Sukumaran et al. 2015). To study whether human CDK5RAP2 has a similar activity, we first investigated whether there exists a similar interaction in the human system and then analyzed the Hippo pathway in WT and patient fibroblasts.

**Fig. 6:**
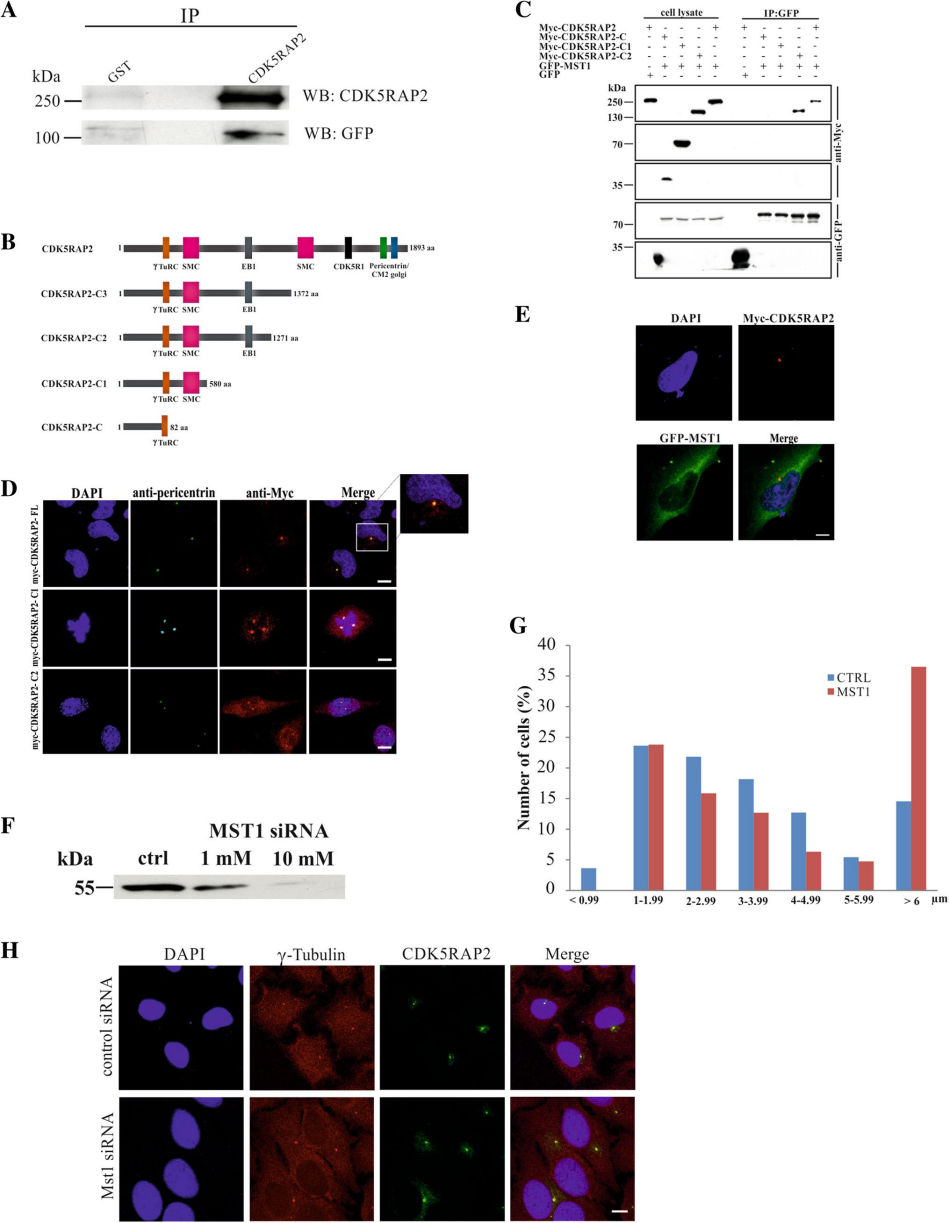
CDK5RAP2 and the link to Hippo pathway components. **a** Co-immunoprecipitation of CDK5RAP2 and GFP-MST1. CDK5RAP2 was immunoprecipitated from HeLa cell lysates expressing GFP-MST1. GFP-MST1 and CDK5RAP2 were detected with GFP-specific mAb K3-184-2 and polyclonal CDK5RAP2 antibodies, respectively. GST-specific polyclonal antibodies were used for control. **b** Schematic of Myc-tagged CDK5RAP2 proteins. The amino acid residues and the domains are indicated. **c** CDK5RAP2 interaction with MST1. Myc-tagged CDK5RAP2 proteins were coexpressed in HEK293T cells with GFP-tagged MST1 and Myc-tagged polypeptides precipitated with Myc antibodies. GFP-MST1 was detected in the precipitates with mAb K3-184-2. GFP was used for control. **d** Immunofluorescence analysis of HeLa cells expressing Myc-tagged CDK5RAP2 proteins. Myc was recognized by mAb 9E10, pericentrin was detected with polyclonal antibodies, nuclei were stained with DAPI. The* boxed area* is enlarged at the* right*. *Scale bar* 10 µm. **e** Immunofluorescence analysis of HeLa cells expressing Myc-tagged CDK5RAP2 and GFP-MST1. Myc antibodies mAb 9E10 detected Myc-CDK5RAP2, GFP-specific mAb K3-184-2 detected GFP-MST1, the centrosome was detected with pericentrin-specific antibodies, DNA was stained with DAPI. *Scale bar* 10 µm. **f** Knockdown of MST1 with siRNA in HeLa cells leads to reduced MST1 protein amounts and an increased centrosome nucleus distance. Detection of the centrosome was with γ-tubulin-specific antibodies. **G** *Bar graph* analysis of the centrosome nucleus distance. The distance from the nucleus was determined using the Leica LAS AF lite program. The number of cells in percent is given. For control 58 cells and for the knock down 66 cells were evaluated. **h** Immunofluorescence analysis of control and MST1 knockdown cells staining for γ-tubulin and CDK5RAP2. Cells treated with 1 nM siRNA are shown. *Scale bar* 10 µm

To probe an interaction between CDK5RAP2 and Hippo pathway components, we expressed GFP-tagged MST1 in HeLa cells and used CDK5RAP2-specific antibodies for immunoprecipitation. GFP-MST1 was found in the immunoprecipitate. GST-antibodies which were used for control did not precipitate the protein (Fig. 6a). Detection of endogenous MST1 in the immunoprecipitate was prevented due to co-migration of MST1 (~55 kDa) with the IgG heavy chain of the antibodies used in the immunoprecipitation. To identify the interacting domain within CDK5RAP2, Myc-tagged full-length CDK5RAP2 and C-terminally truncated CDK5RAP2-C (residues 1–82), –C1 (residues 1–580), –C2 (residues 1–1271) and –C3 (residues 1–1372) were coexpressed with GFP-MST1 in HEK293T cells (Fig. 6b). Myc-CDK5RAP2-C2 harboring the γTURC, SMC and EB1-binding domain was the shortest protein that could precipitate GFP-MST1. GFP alone did not coprecipitate with the CDK5RAP2 proteins (Fig. 6c). The localization of the Myc-tagged truncated proteins was revealed by immunofluorescence studies carried out in HeLa cells. Full-length CDK5RAP2 as well as C1 and C2 were present as a dot near the nucleus colocalizing with pericentrin (Fig. 6d). This is in agreement with previous work by Barrera et al. (2010) who reported that the first 435 amino acids were sufficient for targeting the centrosome. In the C1- and C2-expressing cells, we noted an increased centrosome number which could be due to the overexpression of the protein.

We expanded our studies on the MST1–CDK5RAP2 interaction and carried out an immunofluorescence analysis where we observed that GFP-MST1 was present in the cytosol and also showed an enhanced staining near the nucleus where it colocalized with Myc-CDK5RAP2-C2 detected with mAb 9E10 and pericentrin as centrosomal marker (Fig. 6e). MST1 localization at the centrosome has also been shown in previous work (Hergovich et al. 2009). We then depleted MST1 from HeLa cells using siRNA and studied whether this has an effect on centrosome position, centrosome number and recruitment of CDK5RAP2 to the centrosome. A significant reduction of MST1 levels was observed by western blot analysis (reduction to 83.7% of normal levels with 1 nM siRNA; reduction to 54.6% with 10 nM siRNA; β-actin levels served as control) confirming the knockdown of MST1 (Fig. 6f). The centrosome nucleus distance was enhanced and 36.5% of the cells had the centrosome more than 6 µm away from the nucleus as compared to 14.5% for control cells (Fig. 6g, h). CDK5RAP2 stayed associated with the γ-tubulin-positive centrosome and the typical “Golgi-like” staining was observed (Fig. 6h). The centrosome number was not significantly affected.

**Fig. 7:**
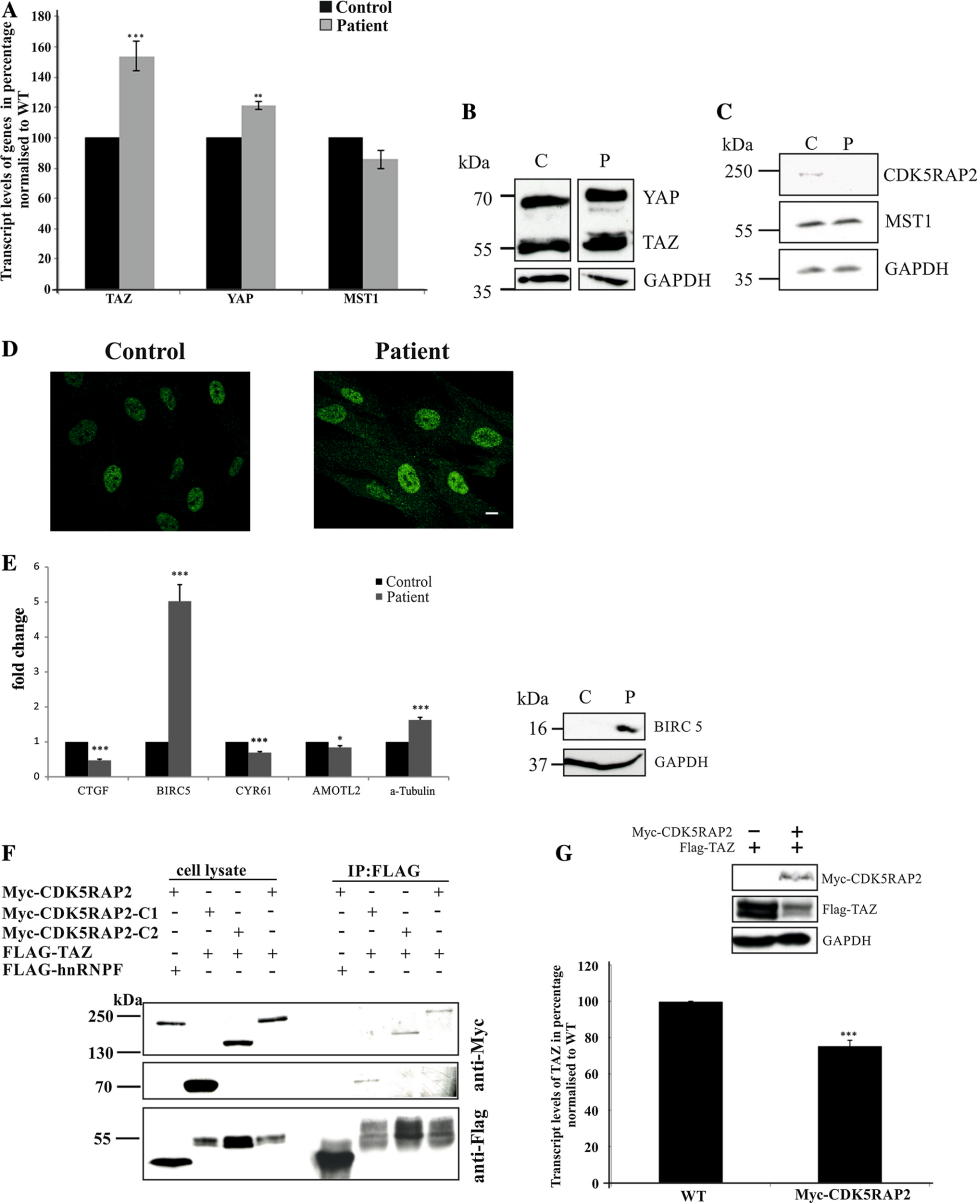
The Hippo signaling pathway is affected in patient fibroblasts. **a** Quantification of the endogenous TAZ (***P < 0.001), YAP (**P < 0.05) and MST1 transcript levels in control and patient fibroblasts by quantitative RT-PCR. **b** YAP and TAZ in whole cell lysates obtained from control and patient cell lysates. Probing was with YAP/TAZ-specific antibodies (YAP ~70 kDa; TAZ ~50 kDa) and GAPDH-specific antibodies (GAPDH ~38 kDa) as loading control. **c** MST1 protein levels appear unaltered in patient cells. Antibodies specific for CDK5RAP2, MST1 and GAPDH were used. **d** YAP and TAZ distribution in control and patient fibroblasts. Immunofluorescence analysis was carried out with YAP/TAZ antibodies. *Scale bar* 10 µm. **e** Hippo pathway genes are downregulated in patient cells as shown by qRT-PCR analysis (left panel) (*P* values, *<0.05, ***<0.001). The increase in BIRC5 transcript levels leads to increased protein amounts (*right panel*). **f** Co-immunoprecipitation assays to study the interaction of CDK5RAP2 with TAZ. Myc-tagged CDK5RAP2 full length, and the C1 and the C2 proteins were tested. HEK293T cells were transiently co-transfected with Flag-tagged TAZ and Myc-tagged CDK5RAP2 proteins. Immunoprecipitation (IP) was done with Flag-specific beads, the resulting blots were probed with mAb 9E10 to reveal Myc-tagged and pAb anti-Flag to reveal FLAG-tagged proteins. FLAG-hnRNPF was used as control. **g** Effect of ectopically expressed Myc-CDK5RAP2 on endogenous TAZ mRNA levels as revealed by qRT-PCR to analyze the transcript abundance of endogenous TAZ in untransfected HEK293T cells (WT) and in cells ectopically expressing Myc-CDK5RAP2 (***P < 0.001). The mRNA reduction leads to decreased protein level (panel above the graph). HEK293T cells were transfected with plasmids coding for Myc-CDK5RAP2 and Flag-TAZ as indicated

Based on these data, we turned to control and patient fibroblasts to explore the Hippo pathway and studied the transcript levels of the core Hippo signaling components TAZ, YAP and MST1. The qRT-PCR studies showed significantly higher transcript levels of TAZ and YAP in the patient fibroblast cells, those of MST1 were not significantly altered (Fig. 7a). The qRT-PCR data were further supported by analyzing the protein levels using pAb YAP/TAZ, which showed concordant increase in the expression levels of the ~70 kDa YAP (twofold, mean of three experiments) and ~50 kDa TAZ (threefold, mean of four experiments) as compared to the control (a representative experiment is shown in Fig. 7b). In support of these findings, overexpression of CDK5RAP2 in HEK293T cells led to reduced amounts of coexpressed Flag-tagged TAZ protein (see below, Fig. 7g). There were no significantly changed MST1 and phosphorylated MST1 protein levels in the patient cells as compared to the control (Fig. 7c and data not shown). In immunofluorescence studies, we observed in control cells a weak staining for YAP/TAZ in the cytoplasm and in the nucleus, patient cells exhibited a slightly enhanced staining in both compartments (Fig. 7d).

### Impact of the Hippo pathway at the transcriptional level

The Hippo pathway acts primarily in an inhibitory fashion on the transcription of genes involved in cell proliferation through inactivating YAP/TAZ. We, therefore, determined the levels of commonly used YAP/TAZ target genes, namely CTGF, BIRC5 (Survivin), CYR61 and AMOTL2, by qRT-PCR and found that in the patient fibroblasts the transcript amounts of all genes were significantly lower as compared to the wild type. The levels of CTGF were most affected and reduced to about half of wild-type levels. The increased levels of YAP and TAZ apparently are not leading to increased levels of their downstream targets (see also discussion). An exception was BIRC5 for which we found a strong increase (~fivefold) (Fig. 7e). The protein levels of BIRC5 were also significantly increased. In western blots, we easily detected the protein in patient cell lysates whereas in the control nearly no signal was seen (Fig. 7e, right panel). The data suggest that in fibroblasts the transcriptional regulation by the Hippo pathway functions in the absence of CDK5RAP2.

### CDK5RAP2 interacts with TAZ

We also tested whether CDK5RAP2 can interact with TAZ and co-transfected HEK293T cells with Myc-CDK5RAP2, Myc-CDK5RAP2-C1 and -C2 and FLAG-TAZ and precipitated FLAG-TAZ using FLAG-trap beads. In the precipitate, we detected Myc-CDK5RAP2, CDK5RAP2-C1 and -C2 indicating that the binding site is located between residues 1 and 580 which encompass the γTuRc domain (Fig. 7f). It thus is different from the binding site of MST1 which is precipitated by C2 as the shortest protein. qRT-PCR analysis using RNA obtained from HEK293T cells overexpressing Myc-CDK5RAP2 showed a highly significant decrease in the transcript levels of TAZ in relation to the control (graph in Fig. 7g). The protein levels were also lower (Fig. 7g, panel above graph). The findings thus are in accordance with the results from the patient samples where we had observed an increase in the transcript levels of TAZ upon CDK5RAP2 deficiency (Fig. 7a).

## Discussion

In two siblings suffering from MCPH, we identified a novel mutation in *CDK5RAP2* leading to a strong reduction or absence of the full-length protein in fibroblasts as demonstrated by various methods. Immunofluorescence analysis showed staining with CDK5RAP2 antibodies in some but not all patient cells. Moreover, the staining in CDK5RAP2-positive cells was reduced and in cells that reacted with the antibodies the Golgi association was not detected. In western blots no signal was detected; however, the signal obtained in control cells was not very strong either. qRT-PCR analysis revealed the presence of reduced amounts of mRNA. Taken together, the mutation in the patient does not lead to a complete loss of CDK5RAP2.

Previously, Issa et al. (2013) investigated immortalized lymphoblasts derived from patients carrying the mutation p.Arg1481*. They mainly carried out immunofluorescence studies and observed mitotic spindle defects and centrosome disorganization. CDK5RAP2 protein was not detectable in these cells. We analyzed the effect of the mutation p.Arg1372* on cell size, migration and polarization. These functions can be associated with the centrosome and a functioning microtubule system. On the other hand, they are also regulated by the Hippo signaling pathway (Varelas 2014). The mutant cells had a higher speed of migration as compared to the wild type, were able to polarize and were proficient to close the wound in a scratch assay. This occurred faster than in the control and could be due to the enhanced speed of migration. The cells also harbored numerous nuclear alterations such as number alterations, presence of micronuclei and distorted nuclei. The nucleus–centrosome distance was also affected in the patient cells where in only ~74% of cells the centrosomes were located close to the nucleus whereas in controls it was over 90%. Similar nuclear and centrosomal defects have been described for MCPH-mutant cells which carried mutations in *CEP135* and *CDK6* (Hussain et al. 2012, 2013).

The microtubule network in mitotic cells appeared more delicate and astral microtubules were less pronounced or absent. Studies by Fong et al. (2008) had shown that CDK5RAP2 loss inhibited centrosomal microtubule nucleation and led to the formation of anastral mitotic spindles although there are also conflicting reports (Lucas and Raff 2007). This activity of CDK5RAP2 is based on the presence of the γTURC domain which mediates γTURC attachment and nucleation of microtubules. Reduced levels of CDK5RAP2 or its absence might, therefore, be responsible for our observations. At the protein level, we observed a significant increase in tubulin in interphase which was paralleled by increased transcript levels. In summary, the analysis of the patient cells yielded data that seem to agree with those obtained from the analysis of other MCPH cells with respect to centrosomal and nuclear abnormalities.

We report further a connection of CDK5RAP2 to the Hippo pathway by showing that the protein can interact with two components of the pathway, the kinase MST1 and the transcriptional regulator TAZ. In the mutant fibroblasts we found increased YAP/TAZ levels. This did, however, not result in increased transcription of YAP/TAZ target genes (see also below). In fact their transcript levels were significantly reduced. For the patient situation this might be important for cell proliferation. An exception was the BIRC5 gene for which we found strongly increased transcript levels accompanied by increased protein levels. BIRC5/Survivin might account for some properties of the patient fibroblasts. BIRC5 has several roles. It promotes cell proliferation and prevents apoptosis. It was also identified as component of a chromosome passenger protein complex (CPC), a complex which is essential for chromosome alignment and segregation during mitosis. CPC has different locations. It is present at the centromere during prometaphase and at the midbody during cytokinesis and is involved in the organization of the spindle by associating with polymerized microtubules. In addition to the Hippo pathway, BIRC5 is regulated by other developmental signaling pathways such as the Wnt/β-catenin, the Hedgehog and the Notch pathway (Altieri 2015). In the murine embryonic brain, conditional deletion of survivin leads to apoptosis of neuronal precursor cells in the CNS and in newborn mutants a marked reduction in the size of the brain was observed (Jiang et al. 2005).

A link between an MCPH protein and the Hippo pathway is very appealing. During the last couple of years it has become clear that Hippo signaling regulates stem cell and progenitor pools in mammals. This has mainly been studied for the intestine but it appears to be relevant for other tissues as well (Camargo et al. 2007; Cao et al. 2008). For mammalian brain development, an involvement of the Hippo pathway has been described and in particular a role for YAP/TAZ in the regulation of self-renewal and expansion of tissue progenitor cells has been proposed. An important component in this event is the tumor suppressor neurofibromatosis 2 (NF2, merlin) which limits the expansion of neural progenitor cells (NPCs) in the mammalian dorsal telencephalon through suppression of YAP activity as reported for mouse (Lavado et al. 2013, 2014). It was also shown that knockdown of LATS1/2 or expression of dominant-negative MST2 caused neuroepithelial proliferation. The authors, therefore, concluded that in vertebrates MST1/2 and LATS1/2 regulate neural progenitor proliferation and survival through inhibiting the activity of YAP (Cao et al. 2008).

On the other hand, MST1 has also a centrosomal role as it was recently reported that MST1 signaling controls centrosome duplication. It was found that overexpression of MST1 caused centrosome overduplication and RNAi depletion led to impaired centriole duplication. This effect was due to MST1 kinase activity since an overexpressed kinase dead MST1 did not show a similar effect (Hergovich et al. 2009). We found that CDK5RAP2 binds MST1 and could be the anchoring point for the kinase which then affects the centrosome. The centrosomal defects which we observed might, therefore, be a consequence of either loss of CDK5RAP2 in the patient or occur upon overexpression of CDK5RAP2 mutant proteins which no longer can anchor MST1 at the centrosome due to loss of the binding domain. Our observation that knockdown of MST1 leads to increased centrosome nucleus distance would support this assumption. Interestingly, the amino acids of CDK5RAP2 that are responsible for the interaction with MST1 are missing in several MCPH3 mutations which gives rise to shortened proteins (Kraemer et al. 2011). A connection to the centrosome was also reported for the closely related MST2 and the Hippo pathway component Salvador which both act in localizing Nek2 kinase to the centrosome. In that particular work, the focus was on the MST2 knockdown effect on centrosome separation (Mardin et al. 2010).

Although we have found increased YAP/TAZ levels, we did not observe enhanced target gene transcription. Increased nuclear YAP is not necessarily a sign for increased YAP activity. This was concluded from experiments in which a Yap (S112A) knock-in mutation in the endogenous Yap locus of mice was generated which is an activating mutation and which resulted in normal mice (Chen et al. 2015). S112 phosphorylation is required for cytoplasmic translocation and binding to 14-3-3. We also found an interaction of CDK5RAP2 with TAZ. The binding site appears to be different from the one of MST1 and the implications of this interaction are not clear at present. How the enhanced YAP/TAZ transcript levels are achieved in the patient cells is unclear. There exist only few studies on the regulation of YAP gene expression. So far β-Catenin/TCF4, miRNAs, ETS transcription factors and cJUN have been shown to be involved (Liu et al. 2010; Konsavage et al. 2012).

It was reported that loss of Mst1/2 or Lats1/2, or activation of YAP-TEAD in neural progenitor cells leads to a marked expansion of neural progenitors, partially due to upregulation of cell cycle re-entry and stemness genes, and a concomitant block to differentiation by suppressing key genes. Conversely, YAP/TAZ loss of function results in increased cell death and precocious neural differentiation (Cao et al. 2008). Furthermore, bone marrow-derived mesenchymal stem cells depleted of TAZ show decreased osteogenesis (Hong et al. 2005). In addition, as mentioned previously, YAP and TAZ proteins are important in brain development (Lavado et al. 2013). In neuronal cells, loss of CDK5RAP2 might lead to insufficient TAZ-dependent proliferative signaling under certain conditions leading to a reduced size of the brain.

**Fig. 8:**
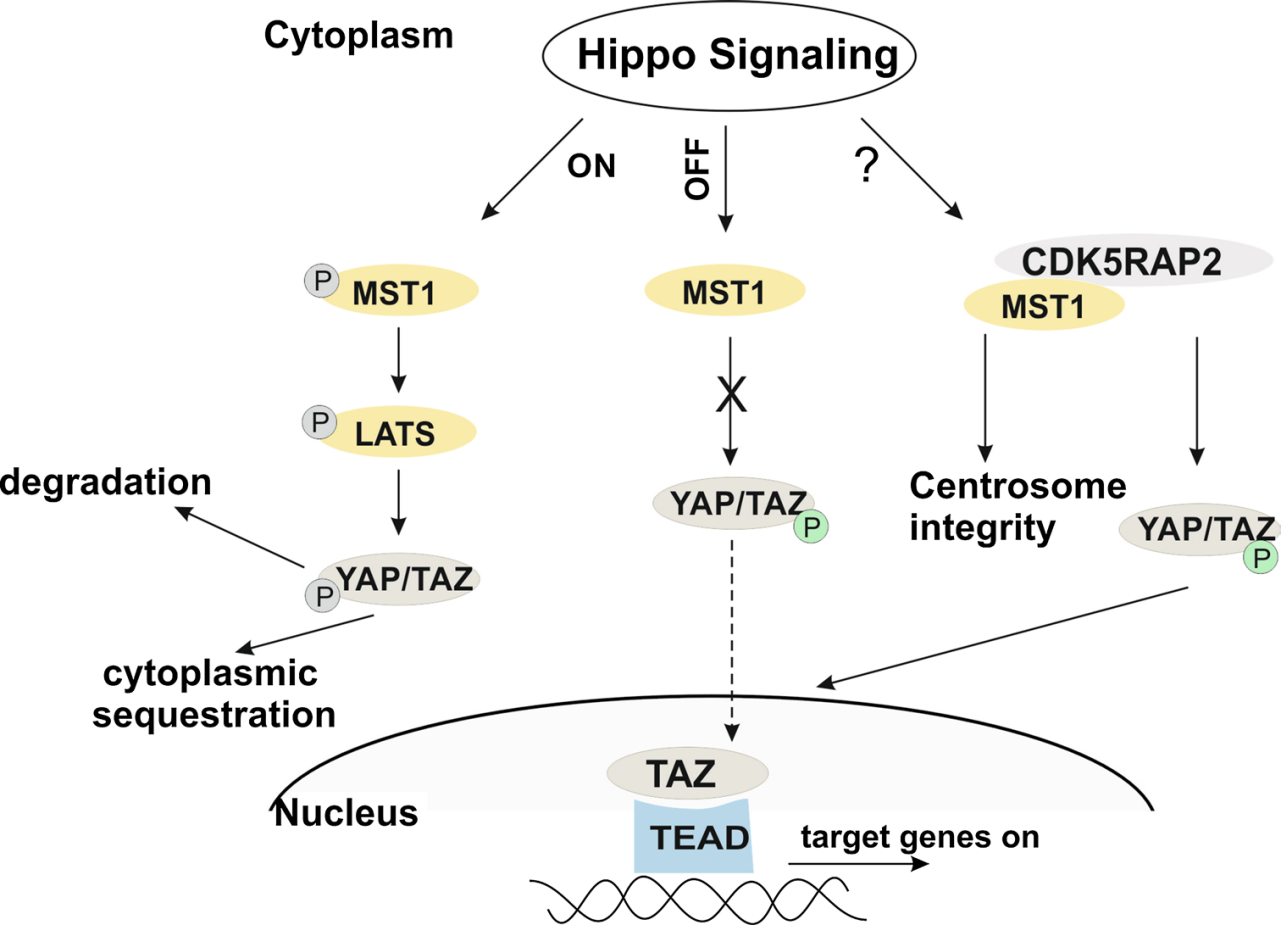
Model for CDK5RAP2 integration in Hippo signaling. *Left* overview of the Hippo signaling pathway. *Right* CDK5RAP2 interaction with MST1 and its impact. *Green color* for P residue indicates phosphorylation (color figure online)

Our analysis was carried out in primary fibroblasts and it is not clear whether the status of Hippo signaling resembles the one in the embryonic brain. In addition, it has been revealed that cell polarity, cell adhesion, cell contacts and mechanical cues, soluble factors and also the metabolic status of the cells are impacting on the Hippo pathway and they all can vary in different situations (Yu et al. 2015). Combining all findings, we propose a dual role for CDK5RAP2 and Hippo pathway components. In neuronal cells, loss of CDK5RAP2 leads to insufficient YAP/TAZ-dependent proliferative signaling under certain conditions resulting in reduced size of the brain which is in agreement with our findings of reduced expression of YAP/TAZ target genes in patient cells (Fig. 8). This idea is also supported by the observation that there is a significant reduction in brain size in MCPH patients with a mutation in *CDK5RAP2* leading to a non-functional protein (Kraemer et al. 2011). We presume that there is a requirement for perfectly balanced amounts of YAP/TAZ in neural stem and progenitor cells which are involved in controlling correct expansion of the progenitor pool and timely differentiation and that this might be ensured by a crosstalk between CDK5RAP2 and the Hippo pathway. Second, CDK5RAP2 provides a physical binding site for MST1 (and also TAZ) (Fig. 8). This interaction is important for the integrity of the centrosome with its important roles in neurogenesis (Chavali et al. 2014).

## Electronic supplementary material

Below is the link to the electronic supplementary material.

Supplementary material 1 (DOCX 14 kb)
