# Supplementary material for: CDK5RAP2 interaction with components of the Hippo signaling pathway may play a role in primary microcephaly
Source: Mol Genet Genomics. 2016 Dec 21;292(2):365–83. doi: 10.1007/s00438-016-1277-x (PMC5357305; doi:10.1007/s00438-016-1277-x)
Supplement: Supplementary file 1 — Supplementary material 1 (DOCX 14 kb) [file 438_2016_1277_MOESM1_ESM.docx]

**Supplementary Material**

**Supplementary Table 1**: Primer sequences

| Primer name | Sequence 5‘-3‘ |
| --- | --- |
| CDK5RAP2-Exon27-F | CCTGGCTTGGGATTTTAGC |
| CDK5RAP2-Exon27-R | TCAAATGTCACCACACTCTGC |
| CDK5RAP2 5’-end F | GTTTGTGAGCAGTGTGAGCA |
| CDK5RAP2 5’-end R | TGGTGACCTCTGCCTTCATT |
| CDK5RAP2 3’-end F | CGCTCACAAAACCTCACCAA |
| CDK5RAP2 3’-end R | CTCCAGTCTCTCCCTCTCCT |
| TAZ F | TCCCAGCCAAATCTCGTGATG; |
| TAZ R | AGCGCATTGGGCATACTCAT |
| MST1 F | TGGGTGAATTCTGGATGGCT |
| MST1 R | GACTGTCAAGAGAGCCCTTC |
| YAP F | CCCAGACTACCTTGAAGCCA |
| YAP R | CTTCCTGCAGACTTGGCATC |
| CTGF-F | GCACCAGCATGAAGACATACCGAGCTA |
| CTGF-R | TAATGGCAGGCACAGGTCTTGATGAAC |
| BirC5-F | GACTTGGCCCAGTGTTTCTTCTGCTTC |
| BirC5-R | GCTTCTTGACAGAAAGGAAAGCGCAAC |
| Cyr 61-F | TCAAACAACTTCATGGTCCCAGTGCTC |
| Cyr 61-R | TAAACCTGACTGGTTCGGGGGATTTCT |
| Amotl 2-F | CGACATGACCAAGTGGGAGCAGAAGTA |
| Amotl 2-R | TGGAGCACCTTTAACCTGCTTTCCATC |
| α-Tubulin-F | TACCCCCGCATCCACTTCCCCCTGGCCAC |
| α-Tubulin-R | GGGCACCAATCTACAAACTGGATGGT |
